# Reliability of Ulnar Nerve Sensation Tests in Patients with Cubital Tunnel Syndrome and Healthy Subjects

**DOI:** 10.3390/diagnostics12102347

**Published:** 2022-09-28

**Authors:** Tomasz Wolny, César Fernández-de-las Peñas, Arkadiusz Granek, Paweł Linek

**Affiliations:** 1 Institute of Physiotherapy and Health Sciences, Musculoskeletal Elastography and Ultrasonography Labolatory, The Jerzy Kukuczka Academy of Physical Education, Mikołowska 72A, 40-065 Katowice, Poland; 2 Department of Physical Therapy, Occupational Therapy, Rehabilitation and Physical Medicine, Universidad Rey Juan Carlos, 28032 Alcorcon, Madrid, Spain; 3 Hospital of the Ministry of Interior and Administration, 25-316 Kielce, Poland

**Keywords:** two-point discrimination test, sensory threshold test, cubital tunnel syndrome, healthy volunteers, ulnar nerve

## Abstract

Static two-point discrimination (2PD) and Semmes–Weinstein monofilament (SWM) tests are commonly used to evaluate sensory disorders in the hand. The aim of this study was to evaluate the reliability of 2PD and SWM tests in the ulnar nerve innervation area in patients with cubital tunnel syndrome (CuTS) and healthy individuals. This was a two-group repeated-measures inter-rater and intra-rater reliability study. Twenty-one patients with CuTS and 30 healthy adults participated. The static 2PD test was performed using a standardized Dellon discriminator, whereas the SWM test was conducted using TOUCH TEST monofilaments. Two examiners performed both tests at the hypothenar eminence and the fourth and fifth digits (ulnar nerve innervation hand territory). First, examiner A conducted three series of 2PD and SWM tests twice with a 15-min rest period (within-day intra-rater reliability). Next, examiner B repeated the same examination 5 min after (inter-rater reliability). Examiner A conducted the same examination 7 days after (between-day intra-rater reliability). For single measurements, the inter-rater reliability and within-day intra-rater reliability in the 2PD was at least 0.81 in patients with CuTS or healthy subjects. The between-days intra-rater reliability for a single measurement varied from 0.56 to 0.95 in CuTS patients and healthy subjects. The between-days intra-rater reliability for mean value from three measurements was above 0.80. The kappa for SWM was above 0.8 and *the percentage of agreement was at least 90%* for all sessions and trials. In conclusion, the 2PD and SWM tests are reliable for assessing sensation in the ulnar nerve innervation area of the hand in patients with CuTS and healthy subjects.

## 1. Introduction

Several conditions of the peripheral and central nervous systems exhibit sensation loss [1,2,3]. Reliable sensory tests are an essential component of neurological examination. Sensation testing helps to detect sensory deficits, which may be useful in the diagnosis and prognosis of painful dysfunctions [4]. It is also helpful in monitoring the progress of an ongoing intervention and its treatment effects [4,5]. Static two-point discrimination (2PD) and Semmes–Weinstein monofilament (SWM) tests are the most popular tests used to evaluate sensory disorders in the hand and have applications in the diagnosis of numerous diseases such as multiple sclerosis, diabetes, stroke, carpal tunnel syndrome, and cubital tunnel syndrome [2,3,6,7,8,9]. The static 2PD test is used to evaluate the innervation density of slowly adapting nerve fibers [10]. An inexpensive and readily available tool is used to perform static 2PD tests, and some authors consider this test a gold standard in neurosensory examinations of the hand [11]. The SWM test, on the other hand, is used to evaluate the threshold of tactile sensitivity due to pressure applied with a specific force [12,13]. For this test, Semmes–Weinstein monofilaments are used, which are also an inexpensive and accessible tool and are considered the best method for assessing tactile sensitivity threshold [14,15]. 

Despite the widespread use of 2PD and SWM tests in clinical practice, the reliability of these tests is somewhat controversial. Some authors have confirmed the reliability of 2PD in post-stroke patients [16], carpal tunnel syndrome patients [4,17], patients with digital nerve damage [11], and children with hemiplegia [18]. For SWM, high reliability has been observed in patients with burn scars [19], Charcot-Marie-Tooth disease [20], and after a stroke [21]. However, there are studies indicating a low intra- and interobserver reliability of both tests (2PD and SWM) in healthy (asymptomatic) subjects [5,22] and those undergoing digital nerve repair [1]. This discrepancy suggests that 2PD and SWM are perhaps reliable only in specific clinical entities, which may cast doubt on their usefulness in the management of the diseases involving sensory disorders in the hand. Therefore, it seems reasonable that the use of 2PD and SWM in clinical practice or research should be preceded by the evaluation of the reliability of these tests for a specific disease entity. 

Cubital tunnel syndrome (CuTS) is the second most common peripheral neuropathy of the upper limb with an estimated prevalence in 2–6% of the population [23]. Some authors have used the 2PD and SWM tests to evaluate the efficacy of therapies for CuTS [8,24,25]. Of course, it should be emphasized at this point that the ulnar nerve is primarily a nerve consisting of motor fascicles. Therefore, studying motor function and nerve conduction is paramount. However, in the comprehensive patient assessment needed for planning physiotherapy and assessing the effectiveness of therapy, the study of different types of sensation also appears to be useful. However, to the best of our knowledge, no study has evaluated the reliability of 2PD and SWM tests in patients with CuTS. We believe that, based on the current knowledge, such a study is warranted and may potentially influence the diagnostic, prognostic, or rehabilitation procedures of patients with CuTS. The reliability and agreement of 2PD and SWM will be important to ensure research and measurement quality in future studies assessing hand sensation in individuals with CuTS. Therefore, the purpose of this study was to evaluate the intra-rater and inter-rater reliability of the 2PD and SWM tests in the ulnar nerve innervation area in healthy subjects and patients with CuTS.

## 2. Methods

### 2.1. Study Design 

This was a two-group repeated-measures design study including healthy subjects and patients with diagnosed CuTS. The study was conducted in a medical outpatient clinic located in Poland. Two examiners with more than 10 years of professional experience in using 2PD and SWM performed the measurements. In healthy subjects, the dominant and non-dominant hands were evaluated in a random order. In CuTS patients, only the symptomatic side was evaluated. One of the examiners (A) was blinded since he did not know the purpose of the research being conducted and did not know whether he was examining a healthy or symptomatic individual. This examiner participated in the entire study (baseline examination, after 15 min, and after 7 days). Both tests (2PD and SWM) were performed in the innervation area of the ulnar nerve at three sites: the hypothenar eminence and the tips of the fifth and fourth digits on the ulnar side. 

**Figure 1:**
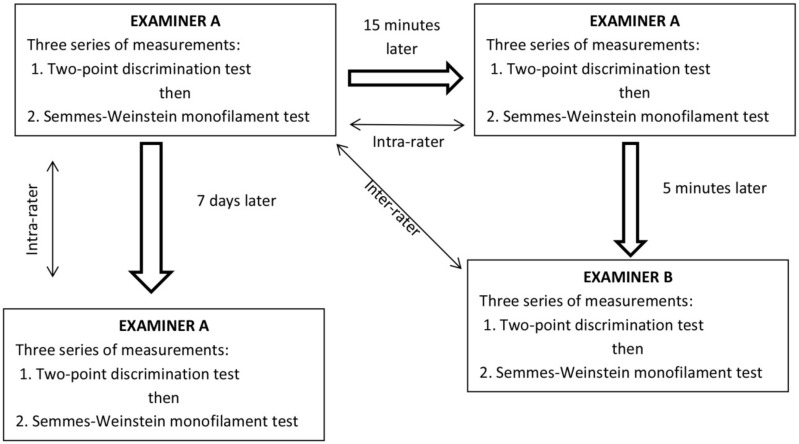
Structure of measurements.

We assessed within-day and between-days intra-rater reliability, as well as inter-rater reliability in both healthy individuals and patients with CuTS (Figure 1). First, examiner A performed three series of the 2PD test followed by another three series of the SWM test in the same order. After a 15-min break, examiner A repeated the same procedure (within-day intra-rater reliability). Then, after a 5-min break, examiner B performed three series of the 2PD test followed by three series of the SWM test in the same order on the same subject (inter-rater reliability). To assess the between-day intra-rater reliability of both tests (2PD and SWM), examiner A conducted the tests following the same procedure 7 days after. 

All participants were informed about the study protocol. Written informed consent was obtained from all participants before collecting any data. All study procedures were performed according to the Declaration of Helsinki of 1975, revised in 1983. The study was approved by the Bioethics Committee for Scientific Research of the Jerzy Kukuczka Academy of Physical Education in Katowice (No. 8/2019, 14 September 2019). 

### 2.2. Participants

Patients with CuTS presenting to an outpatient clinic for physiotherapy management from June 2022 to August 2022 were recruited for the eligibility criteria. The inclusion criteria for subjects with a medical diagnosis of CuTS were clinical symptoms of ulnar peripheral neuropathy (pain, numbness, or tingling, and sensory disturbances of ulnar nerve innervation), and below-normal nerve conduction (motor fiber conduction < 49.3 m/s). In each case, the diagnosis was made by the physician, and the normative values for nerve conduction were determined by the laboratory performing the test. The exclusion criteria for those with CuTS included previous upper extremity surgery, current steroid and nonsteroidal anti-inflammatory drug therapy, cervical radiculopathy, carpal tunnel syndrome, diabetes, and rheumatic diseases.

Healthy volunteers were recruited from individuals accompanying the patients who agreed to participate. The inclusion criteria for healthy subjects were good general health status and the absence of symptoms indicative of ulnar nerve neuropathy (pain, numbness, or tingling in the ulnar nerve innervation area). In addition, healthy participants were excluded if they presented any conditions that might cause sensory disturbances (e.g., diabetes). 

### 2.3. Protocol

**Figure 2:**
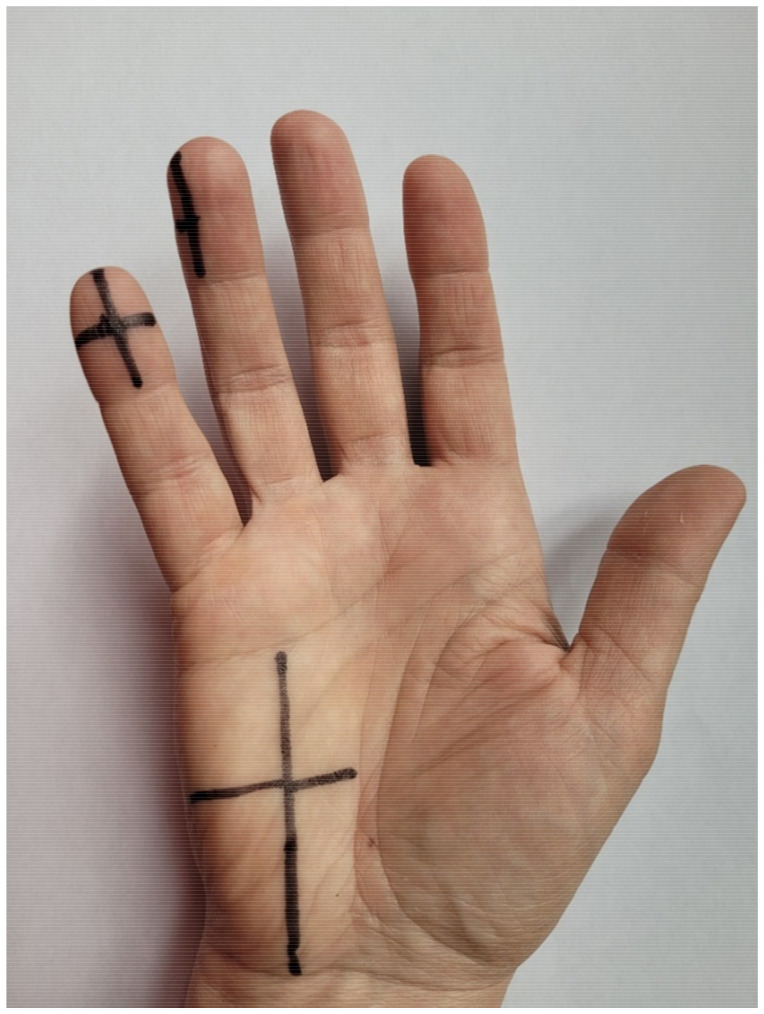
Longitudinal and transverse lines to standardize the measurements.

To improve reliability, longitudinal and transverse lines on the hypothenar eminence and the fourth and fifth digits were drawn to standardize the measurement site for both static 2PD and SWM tests (Figure 2). Each measurement was taken at the intersection of these lines. A caliper and ruler were used to plot the lines. First, the width of the hypothenar eminence was calculated by measuring the distance between the deep palmar arch and the lateral edge of the hand and divided in half to mark this point. Next, the distance between the distal carpal transverse groove and the distal palmar transverse crease was measured and the obtained value was also divided in half to mark the point. Next, using a ruler, two perpendicular lines were drawn to obtain the location of the measurement. Similarly, lines were drawn on the tip of the fifth digit by measuring the width and length of the tip with a caliper. In this case, perpendicular lines were also drawn, with the point of intersection being the measurement location. For the fourth digit, the measurement methodology was the same as for the fifth, except that the vertical line was drawn at a fourth of the tip on its ulnar side (Figure 2). The point where the two lines intersected was where both tests (2PD and SWM) were performed as follows.

During both 2PD and SWM tests, the examiner and the subject sat facing each other. Both arms of the participant were placed along the torso, whereas the forearms (in supination) and hands (palms up) were placed on the table. The study protocol was explained to the subject before the examination began. A training session was then conducted to ensure the participant understood the study protocol. The subject could then see and feel the tactile sensations after touching the hypothenar eminence with a discriminator (single spike) and monofilament (thickest spike). For the examination, a special screen was used so that the subject could not see their hands during the examination.

**Figure 3:**
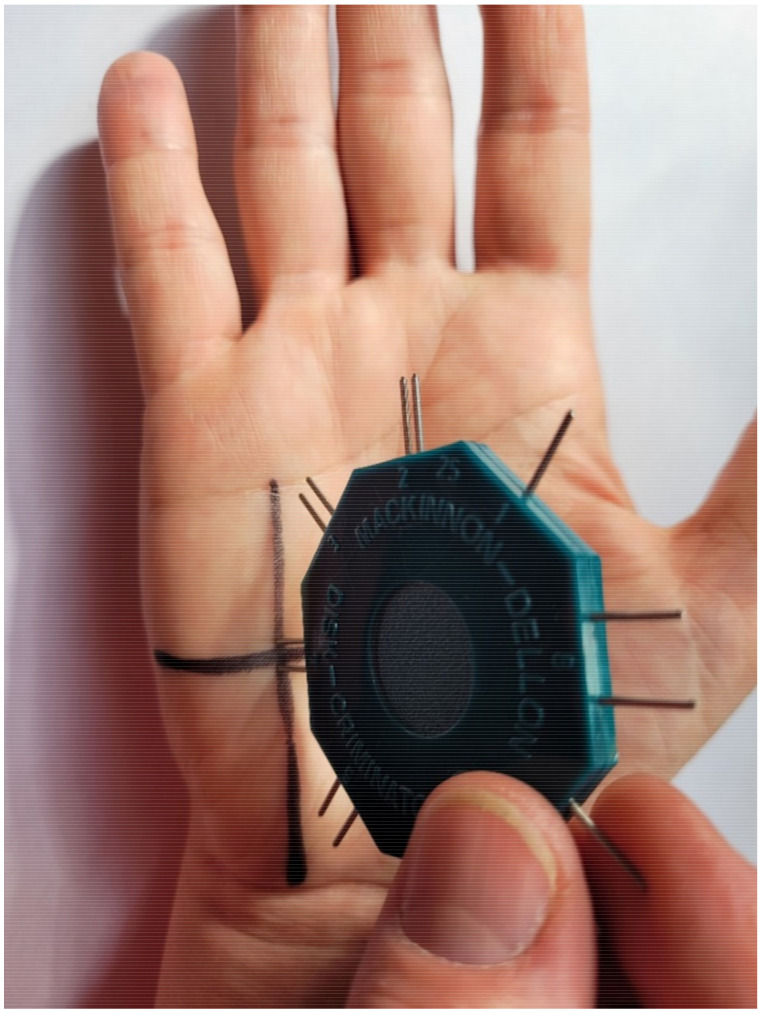
Methodology of testing 2PD.

A standardized Dellon discriminator (Baseline Discrim-A-Gon Discriminator) was used to test static 2PD sensation. This device consists of two plastic disks, each with metal spikes located at specific distances from each other ranging from 2 mm to 15 mm, and one single spike. The test began with the hypothenar eminence, followed by the fourth and fifth digits. The discriminator spikes were applied at the intersection of the marked lines along a vertical line perpendicular to the area tested, while making sure that both spikes touched the test area (Figure 3). The discriminator was applied to the skin without additional pressure (the weight of the device was sufficient for the subject to feel the stimulation). The stimulation time ranged from 3 to 5 s. The measurement locations were randomly touched with one or two discriminator spikes, and the patient was asked to respond “one” if they felt one point or “two” if they felt two points. The shortest distance between discriminator spikes for which the subject provided a “two” response in 3 consecutive measurements was recorded in millimeters and used in the main analysis [4,16]. 

**Figure 4:**
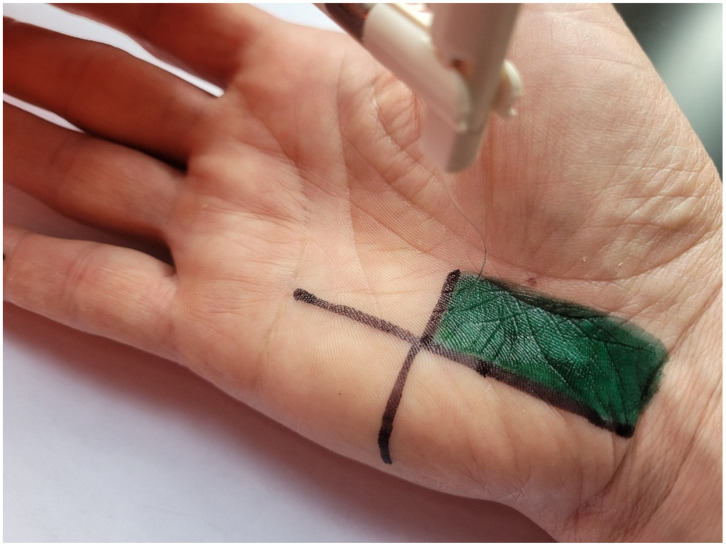
Methodology of testing SWM.

The TOUCH TEST device (North Coast Medical, Inc., Morgan Hill, CA, USA) was used for the SWM test. The device consists of five monofilaments calibrated to produce a specific force in grams: green (size 2.83), 0.07 g; blue (size 3.61), 0.4g; purple (size 4.31), 2.0 g; pink (size 4.56), 4.0 g; and red (size 6.65), 300 g. The monofilament was applied perpendicularly to the measurement location so that a slight deflection occurred. Pressure was maintained from 1 to 3 s. The test began with the thinnest monofilament by randomly touching the crossing point of the previously marked lines on the hypothenar eminence, and the fourth and fifth digits (Figure 4). The subject’s task was to verbally indicate the location of stimulation, i.e., hypothenar eminence, fourth digit, or fifth digit. The thinnest monofilament of three measurements that the subject indicated during stimulation was recorded, and this value was used in the analysis [14,25] in which the following scale was adopted: 0—no sensation during stimulation; 1—0.07 g monofilament; 2—0.4 g monofilament; 3—2.0 g monofilament; 4—4.0 g monofilament; and 5—300 g monofilament [20]. The scale was used in order to calculate the kappa statistic. 

### 2.4. Statistical Analysis

For the SWM test, Cohen’s kappa and the percentage agreement were calculated. For the 2PD test, ICC type 3 (3.1 for single measurement and 3.3 for mean value from three measurements); ICC type 2 (2.1 for single measurement and 2.3 for mean value from three measurements); Bland and Altman (BA) test; and the standard error of measurement (SEM = SD × √(1 − ICC)) were calculated.

The ICC was interpreted as follows: below 0.40 (poor reliability); 0.40–0.59 (fair); 0.60–0.74 (moderate); and above 0.74 (excellent reliability) [26]. The BA test was only used to find potential biases between the two measures. The BA plots with limits of agreement were not included because the sample size was not large enough (greater than 50 participants) to allow the limits of agreement to be estimated properly [27]. The kappa was interpreted as follows: less than 0, poor; 0.00 to 0.20, slight; 0.21 to 0.40, fair; 0.41 to 0.60, moderate; 0.61 to 0.80, substantial; and 0.81 to 1.00, almost perfect [28]. Data were analysed using STATISTICA 13 PL (Statsoft, Tulsa, OK, USA), IBM SPSS version 21 (IBM Corp., Armonk, NY, USA) and Excel (Microsoft Corporation, Redmond, WA, USA) software.

## 3. Results

From 26 patients screened for the eligibility criteria, five were excluded because of steroid and nonsteroidal anti-inflammatory drug therapy (three persons) and diabetes (two persons). Finally, 21 patients with CuTS satisfied all the criteria and agreed to participate. Similarly, from 32 potentially healthy individuals screened, a total of 30 volunteers were finally included. Two people were excluded because they were under diagnosis with suspected diabetes. The characteristics of the subjects are presented in Table 1.

### 3.1. Semmes–Weinstein Monofilament (SWM) 

The kappa for SWM was almost perfect (all trials above 0.81) for all sessions in CuTS (Table 2) and healthy (Table 3) subjects. The within-day intra-rater reliability ranged from 0.91 to 1.00 in CuTS patients and 0.90 to 1.00 in healthy subjects. Similarly, the between-days intra-rater reliability ranged from 0.92 to 1.00 in patients and 0.90 to 1.00 in healthy subjects. The inter-rater reliability was more similar than the intra-rater reliability in CuTS, ranging from 0.92 to 1.00, but it was slightly lower in healthy subjects, ranging from 0.82 to 1.00.

The percentage of agreement was at least 90% in all trials. The percentage of agreement for within-day and between-days intra-rater reliability ranged from 95.2% to 100% in patients with CuTS (Table 2) and from 93.3% to 100% in healthy subjects (Table 3). The percentage of agreement for inter-rater reliability ranged from 95.2% to 100% in patients with CuTS (Table 2) and from 90% to 100% in healthy subjects (Table 3). 

### 3.2. Static Two-Point Discrimination (2PD)

Overall, for a single measurement, the within-day intra-rater reliability was excellent (all trials above 0.80) and the corresponding SEM was always below 0.55 mm in CuTS (Table 4) and healthy (Table 5) subjects. The mean value from three measurements further improved the reliability in both groups of subjects (ICC over 0.89 and SEM below 0.34).

The between-days intra-rater reliability for a single measurement varied from fair to excellent in CuTS patients and from moderate to excellent in healthy subjects. The between-days intra-rater reliability for mean value from three measurements was excellent, and the corresponding SEM was below 0.66 mm in all subjects. In healthy subjects, a systematic error was seen in the hypothenar measurement on the dominant side. In other measurements the bias was close to 0 without any systematic errors, as the line of equality was in the 95% confidence interval. 

The inter-rater reliability for 2PD was always excellent, and the corresponding SEM was below 0.56 mm. Some systematic errors were detected but the bias was close to 0 (Table 4 and Table 5).

## 4. Discussion

The aim of this study was to evaluate the intra-rater and inter-rater reliability of static 2PD and SWM tests in the ulnar nerve innervation area in patients with CuTS and healthy subjects. We observed that both tests (2PD and SWM) have excellent intra-rater and inter-rater reliability in individuals with CuTS and in healthy subjects from the first measurement. The kappa value for the SWM test ranged from 0.9–1.0 and was nearly perfect, and the percentage agreement was always above 90%. In contrast, the standard error of measurement (SEM) in the 2PD test was always below 0.54 at the 15-min interval between measurements. Therefore, it can be stated that both sensation tests (2PD and SWM) are reliable tools for the evaluation of sensation and its disturbances in the innervation area of the ulnar nerve in both intra-rater and inter-rater evaluations, and this applies to measurements after 15 min and after 7 days. Thus, they can be used in patients with peripheral neuropathies of the ulnar nerve as a diagnostic tool to monitor the therapy and clinical outcomes. 

It should be emphasized that this is the first study to evaluate the reliability of the 2PD and SWM tests in the ulnar nerve innervation area in healthy subjects and CuTS patients. It should also be stressed that in the reliability assessment of both tests (2PD and SWM), a new measurement methodology was presented that involved plotting vertical and horizontal lines after previously measuring the hypothenar eminence and tips of the fourth and fifth digits. This may have contributed to the high reliability, as the exact sites of discriminator and monofilament application were standardized.

The reliability of the 2PD test was first confirmed by Dellon et al. [29] in nerve-injured patients for assessing the sensory sensitivity of the hand. Marx et al. [17] and Wolny et al. [4] obtained similar results to our study for the inter-rater and intra-rater reliability of the 2PD test in individuals with carpal tunnel syndrome. The high reliability of the 2PD test has also been found in leprosy patients [30], traumatic median nerve injuries [10], acute stroke patients [16], and children with spastic hemiplegia [18]. Novak et al. additionally observed a strong correlation between the 2PD test and hand function [10]. However, some studies have suggested that the experience of the researcher is an important factor in achieving high 2PD reliability. A study by Marx et al. [17] involved six researchers, two of whom had no prior experience in performing the 2PD test. The reliability achieved by the experienced researchers was significantly higher (ICC 0.85) compared to those without experience (ICC 0.5). In fact, the important effect of the examiner’s experience on the reliability of 2PD measurement was also found by Moberg [31] in a study of people with tetraplegia. However, some studies have shown a poor reliability of the 2PD test, as demonstrated in a study of patients with digital nerve repair [1] and healthy individuals [5,22]. This suggests that the 2PD test should not be used alone in the quantitative evaluation of the sensory recovery process [1]. In these studies, the low reliability of the 2PD test cannot only be explained by the experience of the researchers [22], because in one of them, the research was also performed by people with experience [5]. Therefore, it appears that the discrepancy in the reliability data in previous studies may be primarily due to methodological differences in conducting the 2PD test or the lack of standardization of the measurement procedure, as we have done in our study. It should be noted, however, that the severity of nerve injury may play a role in the differences of these studies.

Lundborg and Rosén [32] emphasized that, although the 2PD test is the most widely used for assessing sensation after nerve repair, the test procedure has not yet been standardized. In assessing the reliability of SWM, this test, similar to 2PD, does not show complete agreement. Meire et al. obtained high inter-rater and intra-rater reliability of the SWM test in subjects with burn scars and healthy subjects [19]. Similarly, good results were presented by Suda et al. in stroke survivors [21] and in patients with Charcot-Marie-Tooth disease [20]. Using a stepping algorithm (4-2-1), Snyder et al. [33] obtained an acceptable inter-rater and intra-rater reliability of the SWM test in healthy subjects. However, other studies have not confirmed the proper reliability of the SWM test in healthy subjects [5,22] and those with digital nerve repair [1], even showing poor reliability. It is worth noting here that the papers showing the low reliability of the SWM also reported the low reliability of the 2PD test. Thus, it is likely that the different methodological differences [1,5] and, probably, low experience of the researchers in the study by Rozental et al. [21] may explain the low reliability of the SWM test observed. 

The methodological part concerning the measurement procedure of the 2PD and SWM tests was poorly described in the studies by Bulut et al. [1,5] and Rozental et al. [22]. Therefore, the results cannot be replicated to verify the methodology used. In the study by Rozental et al. [22], the researchers had no prior experience in conducting sensory tests, only from video instruction, and after participating in one practice session (the duration of which was not specified) they proceeded to examine the reliability of the 2PD and SWM tests. In contrast, in studies that confirmed the high reliability of 2PD and SWM tests, the researchers provided adequate training under the guidance of an expert [19,21] or already had sufficiently extensive experience in conducting the tests [20]. It is also important to note that in studies where the high reliability of 2PD and SWM tests was observed, the description of the test procedure is very detailed, including the location of monofilament application (to always perform the examination in the same place) [19], the duration of point stimulation [19,21], and the interval time between tests [19,21]. All these methodological issues affect the reliability of 2PD and SWM tests and it is possible that in those studies providing low reliability of 2PD and SWM tests [1,5,22] these variables were not controlled. In our study, the excellent reliability of the SWM test was obtained, which is probably due to the researchers’ experience in conducting the test and the author’s methodology for locating the measurement sites. Stimulation time and intervals between tests were also controlled. 

We should recognize some limitations of the current study. The first is the lack of discriminator force control in the 2PD test and monofilament in the SWM test, which may affect sensory evaluation. Such pressure control would probably be beneficial for standardization of the test; on the other hand, the test apparatus would be more complicated, probably more expensive, and less operative in clinical practice. In contrast, examinations performed by clinicians should be characterized by universal access, short testing times, and simple study methodologies. Our study shows that even without pressure control, excellent reliability can be achieved if the adequate testing methodology is used, and the examiner is experienced in the use of sensation testing. Another limitation may also be the lack of nerve conduction studies in healthy individuals, since there may have been cases of abnormal nerve conduction among the included subjects, albeit they were asymptomatic. Another limitation may be that the research was conducted only by experienced researchers. As previously discussed, this topic shows the significant effect of experience on the results, but we do not know whether the proposed new methodology would allow less experienced clinicians to achieve the same reliability. Finally, it should also be considered that this study only evaluated assessment of the reliability of sensory tests, while the ulnar nerve, as a more motor nerve, should be assessed primarily with motor tests.

## 5. Conclusions

The measurement procedure developed for the present study achieved high reliability of the 2PD and SWM tests in assessing sensation in the ulnar nerve innervation area in healthy subjects and patients with CuTS. Therefore, we recommend the use of 2PD and SWM tests in studies of such populations. It should be stressed, however, that tests should only be performed by people with proper training and experience. 

## Figures and Tables

**Table 1:** Descriptive statistics, mean value (SD).

	Healthy Volunteers (n = 30)	CuTS Patients (n = 21)
Age (years)	40 (11.2)	39.8 (9.2)
Height (cm)	174.8 (11.6)	172.8 (10.8)
Body mass (kg)	78.3 (15.5)	75.2 (14.3)
Gender (numbers)	10 (47.6%) Female	10 (47.6%) Female
Affected side	-	18 (85.7%) Right
NCS (MCV m/s)	-	36.2 (6.13)

NCS—nerve conduction study; MCV—motor conduction velocity.

**Table 2:** Reliability and validity of Semmes–Weinstein monofilament (SWM) test—CuTS patients (n = 21).

		Finger 4	Finger 5	Hypothenar
		For Single Measurement		
Rater A	Mean	2.76	2.8	2.76
Rater B	Mean	2.76	2.81	2.8
Intra-rater reliability Rater A (within-day 15 min)	K ^1^	1.0	1.0	0.91
	P_0_% ^2^	100	100	95.2
Intra-rater reliability Rater A (between-days 7 days)	K ^1^	0.92	1.0	1.0
	P_0_% ^2^	95.2	100	100
Inter-rater reliability (baseline assessment)	K ^1^	1.0	1.0	0.91
	P_0_% ^2^	100	100	95.2

^1^ Cohen’s kappa; ^2^ percentage agreement.

**Table 3:** Reliability and validity Semmes–Weinstein monofilament (SWM) test—healthy volunteers (n = 30).

		Side					
		Dominant (Right)			Non-Dominant (Left)		
		Finger 4	Finger 5	Hypothenar	Finger 4	Finger 5	Hypothenar
For Single Measurement							
Rater A	Mean	1.43	1.46	1.8	1.6	1.53	1.96
Rater B	Mean	1.5	1.47	1.8	1.63	1.56	1.93
Intra-rater reliability Rater A (within-day 15 min)	K ^1^	1.0	1.0	1.0	1.0	0.94	0.90
	P_0_% ^2^	100	100	100	100	96.7	93.3
Intra-rater reliability Rater A (between-days 7 days)	K ^1^	0.93	1.0	0.90	1.0	0.94	0.90
	P_0_% ^2^	96.7	100	93.3	100	96.7	93.3
Inter-rater reliability (baseline assessment)	K ^1^	0.87	1.0	1.0	0.94	0.82	0.95
	P_0_% ^2^	93.3	100	100	96.7	90	96.7

^1^ Cohen’s kappa; ^2^ percentage agreement.

**Table 4:** Reliability and validity of two-point discrimination (2PD)—CuTS patients (n = 21).

		Finger 4	Finger 5	Hypothenar
Rater A	Mean ^1^	6.8	6.81	11.71
	SD ^1^	1.12	1.16	1.61
Rater B	Mean ^1^	6.81	6.8	11.76
	SD ^1^	0.98	0.92	1.3
Intra-rater reliability Rater A (within-day 15 min)	For single measurement			
	ICC_3.1_	0.89	0.85	0.89
	SEM (mm)	0.38	0.40	0.53
	Bias ^2^ (mm)	0.24	0.05	0.05
	For mean value from three measurements			
	ICC_3.3_	0.97	0.94	0.96
	SEM (mm)	0.20	0.22	0.27
	Bias ^2^ (mm)	0.11 *	0.05	0.03
Intra-rater reliability Rater A (between-days 7 days)	For single measurement			
	ICC_3.1_	0.83	0.56	0.67
	SEM (mm)	0.47	0.66	0.79
	Bias ^2^ (mm)	0.05	0.05	0.09
	For mean value from three measurements			
	ICC_3.3_	0.97	0.91	0.81
	SEM (mm)	0.19	0.25	0.53
	Bias ^2^ (mm)	0.09	0.09	0.03
Inter-rater reliability (baseline assessment)	For single measurement			
	ICC_2.1_	0.83	0.87	0.85
	SEM (mm)	0.43	0.38	0.56
	Bias ^2^ (mm)	0.01	0.00	0.05
	For mean value from three measurements			
	ICC_2.3_	0.96	0.88	0.95
	SEM (mm)	0.21	0.30	0.29
	Bias ^2^ (mm)	0.05	0.19 *	0.09

^1^ From all three measurements; ^2^ Bland–Altman Test; * systematic error.

**Table 5:** Reliability and validity of two-point discrimination (2PD) test—Healthy volunteers (n = 30).

		Side					
		Dominant (Right)			Non-Dominant (Left)		
		Finger 4	Finger 5	Hypothenar	Finger 4	Finger 5	Hypothenar
Rater A	Mean ^1^	5.16	4.76	8.3	5.26	5.56	8.16
	SD ^1^	1.14	1.1	2.08	1.28	1.35	1.96
Rater B	Mean ^1^	5.23	4.96	8.5	5.63	5.23	8.17
	SD ^1^	0.97	1.12	2.19	1.15	1.22	1.72
Intra-rater reliability Rater A (within-day 15 min)	For single measurement						
	ICC_3.1_	0.83	0.86	0.94	0.85	0.81	0.95
	SEM (mm)	0.45	0.42	0.51	0.49	0.54	0.43
	Bias ^2^ (mm)	0.13	0.26	−0.27 *	0.01	0.40 *	0.20
	For mean value from three measurements						
	ICC_3.3_	0.92	0.96	0.98	0.95	0.90	0.98
	SEM (mm)	0.27	0.21	0.29	0.26	0.33	0.27
	Bias ^2^ (mm)	0.10	0.12	−0.19 *	0.08	0.16	0.07
Intra-rater reliability Rater A (between-days 7 days)	For single measurement						
	ICC_3.1_	0.61	0.82	0.96	0.83	0.76	0.95
	SEM (mm)	0.66	0.45	0.42	0.51	0.62	0.44
	Bias ^2^ (mm)	0.01	0.17	−0.43 *	0.20	0.43 *	0.03
	For mean value from three measurements						
	ICC_3.3_	0.83	0.93	0.97	0.94	0.86	0.98
	SEM (mm)	0.38	0.27	0.35	0.29	0.39	0.27
	Bias ^2^ (mm)	0.07	0.06	−0.25 *	0.13	0.12	0.03
Inter-rater reliability (baseline assessment)	For single measurement						
	ICC_2.1_	0.82	0.89	0.94	0.88	0.85	0.94
	SEM (mm)	0.45	0.37	0.52	0.43	0.50	0.45
	Bias ^2^ (mm)	0.20	−0.20 *	0.07	−0.37 *	0.33 *	0.01
	For mean value from three measurements						
	ICC_2.3_	0.93	0.96	0.98	0.93	0.94	0.96
	SEM (mm)	0.25	0.20	0.29	0.30	0.25	0.37
	Bias ^2^ (mm)	0.01	−0.13 *	0.13	−0.23 *	0.01	0.09

^1^ From all three measurements; ^2^ Bland–Altman Test; * systematic error.

## Data Availability

Data are available upon reasonable request.
